# Intensity discrimination and neural representation of a masked tone in the presence of three types of masking release

**DOI:** 10.3389/fnins.2023.1102350

**Published:** 2023-05-30

**Authors:** Hyojin Kim, Bastian Epp

**Affiliations:** Auditory Physics Group, Hearing Systems Section, Department of Health Technology, Technical University of Denmark, Lyngby, Denmark

**Keywords:** auditory object, masking, EEG, LAEP, salience, intensity JND, comodulation, interaural

## Abstract

**Introduction:**

Hearing ability is usually evaluated by assessing the lowest detectable intensity of a target sound, commonly referred to as a detection threshold. Detection thresholds of a masked signal are dependent on various auditory cues, such as the comodulation of the masking noise, interaural differences in phase, and temporal context. However, considering that communication in everyday life happens at sound intensities well above the detection threshold, the relevance of these cues for communication in complex acoustical environments is unclear. Here, we investigated the effect of three cues on the perception and neural representation of a signal in noise at supra-threshold levels.

**Methods:**

First, we measured the decrease in detection thresholds produced by three cues, referred to as masking release. Then, we measured just-noticeable difference in intensity (intensity JND) to quantify the perception of the target signal at supra-threshold levels. Lastly, we recorded late auditory evoked potentials (LAEPs) with electroencephalography (EEG) as a physiological correlate of the target signal in noise at supra-threshold levels.

**Results:**

The results showed that the overall masking release can be up to around 20 dB with a combination of these three cues. At the same supra-threshold levels, intensity JND was modulated by the masking release and differed across conditions. The estimated perception of the target signal in noise was enhanced by auditory cues accordingly, however, it did not differ across conditions when the target tone level was above 70 dB SPL. For the LAEPs, the P2 component was more closely linked to the masked threshold and the intensity discrimination than the N1 component.

**Discussion:**

The results indicate that masking release affects the intensity discrimination of a masked target tone at supra-threshold levels, especially when the physical signal-to-noise is low, but plays a less significant role at high signal-to-noise ratios.

## 1. Introduction

Acoustic scenes in everyday life consist of a complex mixture of sounds. Our auditory system can segregate this mixture into a target sound and a noise background, enabling communication in acoustically complex environments. One way to describe this is that the auditory system binds various acoustic features arising from the same source into a sound object or an acoustic stream (Bregman, 1994). As an example of such features, speech shows coherent amplitude modulation patterns across a wide frequency range (Raphael et al., 2007). Previous studies have shown that coherent modulation, or comodulation, is beneficial for the detection of a tone in noise (Hall et al., 1984; Nelken et al., 1999). This suggests that comodulation can be used to group spectral components across a wide range of frequency bands into one masker stream based on the idea that comodulation indicates that these components stem from the same source. Such grouping, or auditory stream formation, can facilitate the segregation of the target signal from the noise and result in enhanced target detection. Similarly, spatial information can also facilitate sound detection. When an acoustic source is lateralized relative to the listeners' head direction, interaural disparities between the ears can be induced. For instance, when the target tone is presented with an interaural phase difference (IPD) between left and right ears while the noise is identical between the ears, target detection performance can be enhanced compared to the case with no IPD (van de Par and Kohlrausch, 1999). Furthermore, the target detection performance can be affected non-simultaneously by either preceding sounds or following sounds (Oxenham and Wojtczak, 2010), indicating a role of temporal contexts in auditory stream formation (Dau et al., 2009; Grose et al., 2009). While previous studies have shown how beneficial these cues are for auditory stream formation, it is unknown how these cues interdependently induce auditory stream formation and enhance the audibility of the target tone.

In psychoacoustics, the rationale behind the use of a detection paradigm is that the introduction of a cue can change the representation of the masked signal, and this change then can be quantified with detection thresholds. For target detection, adding a beneficial cue will improve the internal representation of the signal to be detected and hence increases the internal signal-to-noise ratio of the stimulus, enhancing the detection threshold. An enhancement in detection threshold is considered a “release from masking,” often referred to as “masking release”. Masking release can be quantified as the amount of decrease in the detection threshold by adding beneficial cues. A decrease in detection threshold by comodulation is referred to as comodulation masking release (CMR), and the decrease related to a binaural cue is referred to as binaural masking level difference (BMLD). On top of this, temporal context can also affect masking release. For instance, when the target is preceded by a masking sound, the target detection performance can either be enhanced or worsened (Dau et al., 2005, 2009; Grose et al., 2009). From the perspective of auditory stream formation, masking release can be interpreted as the result of grouping frequency components. For CMR, comodulation can group frequency components with the same modulation pattern, inducing stream formation, and hence support the target separation from the masker. In temporal contexts, where preceding or following sounds exist, priming to bounded frequency components can affect the target stream formation non-simultaneously. Regarding the interplay between the temporal context and comodulation, previous studies showed that CMR can be reduced or increased, depending on the preceding and following maskers (temporal fringe) (e.g., Dau et al., 2005, 2009; Grose et al., 2009), suggesting a cortical mechanism underlying CMR or, at least, the dominance of the temporal context cue over comodulation. However, little is known whether the temporal contexts can affect BMLD as well, and how the combination of various cues would affect the audibility at communication sound levels, or supra-threshold levels.

From a physiological perspective, the auditory system will combine available cues and shape the final neural representation of the target in noise. Therefore, depending on the underlying neural circuits for each sound feature encoding, and their mutual dependence and interaction, the effective contribution of each cue to the target separation from noise can vary. Neural correlates of masking release were found at various stages of the auditory pathway. A physiological neural correlate of CMR was found at the cochlear nucleus (CN) level (Pressnitzer et al., 2001; Neuert et al., 2004). They showed that comodulation can enhance the neural activity of the target tone. This representation is relayed to the inferior colliculus (IC) where the neural representations of the modulated stimuli are further improved (Joris et al., 2004). Las et al. (2005) showed that the neural representation of CMR has a resemblance to the physical attributes of stimuli up to the level of the IC and to auditory cortex. They also showed that from higher stages of the auditory pathway, a new neural representation arises at the medial geniculate body (MGB) as hypersensitive locking suppression, which is further enhanced at the auditory cortex (A1) (Las et al., 2005). Diepenbrock et al. (2017) also found neural correlates of CMR in the IC of guinea pigs. They found that units in the IC can show both a reduced response due to the presence of the masker and an enhanced signal response in the presence of a comodulated masker. They suggest that an enhanced signal presentation at the CN is followed by suppression of IC activity by auditory cortex to reduce the response to the masker.

For IPD encoding, several studies found physiological correlates at the IC (Shackleton et al., 2003, 2005; Zohar et al., 2011). As a neural mechanism, Zohar et al. (2011) proposed the first spike latency encoding, showing that the IPD of the stimulus could be estimated by the first *n* spikes of cells tuned to the preferred IPD. Regarding masking release in temporal contexts, Sollini and Chadderton (2016) showed that priming exposure to noise before the target signal can enhance CMR, and that corresponding neural correlates at A1 exist. In their study, by inactivating A1 regions during the priming periods of noise before the target signal, the unmasking effect by the preceding masker was significantly reduced. However, they also observed a slow adaptation to modulation patterns. Hence, Sollini and Chadderton (2016) concluded that A1 may also play a role in cortical feedback to subcortical regions, and not be exclusively responsible for encoding temporal contexts.

To link behavioral measures and neural responses at the cortical level, Epp et al. (2013) used electroencephalography (EEG) to investigate whether the neural representation of a target tone in noise can be reflected in late auditory evoked potentials (LAEPs). Epp et al. (2013) evaluated auditory evoked potentials (AEPs) as a measure of the internal representation of the target tone in noise in the presence of comodulation and IPD. They assumed that the physical signal-to-noise ratio of the masked tone is enhanced by comodulation and IPD cues along the auditory pathway. They varied the intensities of the tonal component with a fixed masker level. They found that the amplitude of the P2 component of the LAEPs was proportional to the amount of masking release, CMR, and BMLD. The growth function of the P2 amplitude was similar across conditions, despite largely different physical intensities of the target tone. Based on this finding, the follow-up study by Egger et al. (2019) suggested that LAEPs measured at the same sensation levels (e.g., threshold +5, +10 dB, etc.) will evoke the same amplitude of the P2 components regardless of masking release conditions. In addition, they hypothesized that the perceptual quality of a masked signal at various levels above masked thresholds varies gradually. They referred to this perceptual quality as “salience”. They postulated the salience to be a behavioral measure of the neural representation of masked tone. The results showed that the tone at the same levels above the masked threshold evoked similar P2 amplitudes, regardless of the presence of comodulation and IPD cues. However, ratings of the salience measured by a scaling method were not correlated with P2 amplitudes, but were partially consistent with an evaluation of partial loudness of the tone. They speculated that this result may be due to the subjective interpretation of salience and that some listeners might have confused salience with the loudness of the tonal signal. Another study aimed to quantify the benefit of masking release at supra-threshold levels by mapping physical properties the intensity of a sound to the partial loudness of the target in the masker (Verhey and Heeren, 2015). Their results also showed a high variability in ratings across and within listeners. A reason for the high variability in the data might be that the method requires listeners to rate loudness and strongly dependent on listeners' subjective criteria for decision-making. Therefore, a robust measure of the perception of a masked tone in noise at supra-threshold measured is desirable.

Combining all these points, masking release can be considered a result of auditory stream formation by comodulation, IPD, and temporal context. Physiological correlates of each auditory feature have been located broadly along the auditory pathway (CN, IC, A1). What is lacking is how the neural encoding of auditory features is combined along the auditory pathway to induce behavioral outcomes or masking release. Moreover, how relevant masking release is for communication at supra-threshold levels is unclear. Thus, this study aims to answer the following research questions; (a) whether each sound feature encoding occurs independently in a serial manner along the auditory pathway, (b) whether masking release is relevant in communication at supra-threshold sound intensities, and (c) if a combined neural representation of the target signal enhanced by each auditory cue can be measured using EEG at the auditory cortex level.

In the present study, we designed the stimuli to induce masking release by comodulation, IPD, and temporal context. This enabled us to investigate the interaction of these three cues on masked thresholds and at supra-threshold levels. We specifically focused on the interaction of these cues when the target is presented at the intensity levels between a masked threshold and levels relevant to communication. We aimed to estimate the effect of preceding masker and stream formation on CMR and BMLD, both behaviorally and electrophysiologically. We hypothesized that if the effect of preceding stream formation is the result of high-level auditory processing at the level of A1 by modifying the input received from the peripheral auditory system, both CMR and BMLD would be affected by preceding maskers. Because the use of “salience” to quantify the perception of a masked tone above masked threshold is challenging, we used a just-noticeable difference (JND) to quantify the perception and neural representation of the tone in noise at supra threshold levels. From the intensity-JND, we derived a measure referred to as “perceived SNR” (pSNR). We used this measure to describe the perceptual quality of the masked tone at supra-threshold levels, and to correlate this perceptual measure with a neural marker. Here, pSNR is considered a continuous variable reflecting the perception of the masked tone relative to the background, close to and well above the masked threshold, including all possible perceptual qualities. This definition is derived from intensity-JND and hence differs from a binary definition of salience as an attribute of a stimulus component that attracts attention or “pops out” from the background (e.g., Kaya et al., 2020; Soeta and Ariki, 2020). Compared to the attempt to ask for a measure of salience directly, the intensity JND approach might potentially reduce the impact of subjective criteria for judging the perception of the target tone in challenging signal-to-noise ratios. We hypothesized that if the internal neural representation is enhanced by a given amount of masking release, conditions with lower detection thresholds will show smaller intensity JNDs (higher pSNR) at the same physical target tone level than conditions with higher detection thresholds. Lastly, we used the LAEP as a neural measure of the internal representation of the masked tone. We estimated the slope of changes in P2 amplitudes with increased intensities. If P2 amplitudes can reflect the pSNR, we hypothesized that the increment in P2 with increasing tone level would be inversely proportional to the intensity JND and the pSNR would be correlated with P2 amplitudes reflecting the internal SNR.

## 2. Materials and methods

### 2.1. Stimuli

**Figure 1:**
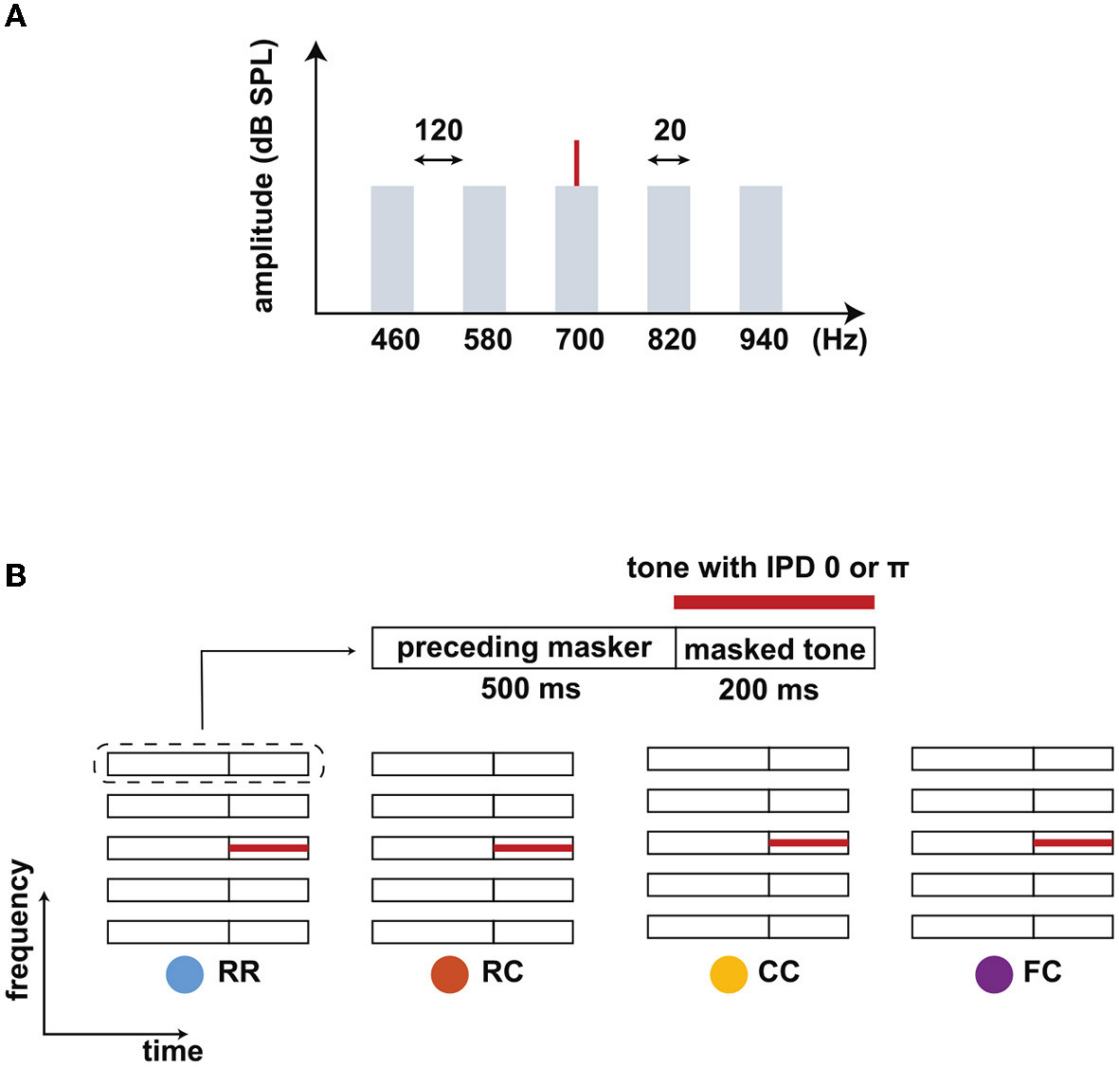
Schematic spetra and spectrograms of the stimuli. **(A)** Spectra of the stimulus. A target tone (700 Hz) was presented with a masking noise consisting of five narrow-band maskers: one band centered at the target tone frequency (center band, CB) and four flanking bands (FBs). The bandwidth of each masker band was 20 Hz, and the frequency spacing between FBs was 120 Hz. The overall level of the noise was set to 60 dB SPL. **(B)** Schematic spectrograms of the stimuli. Each stimulus consists of a preceding masker (500 ms) and masked tone (200 ms). Four types of maskers were used: RR, RC, CC, and FC. The RR was used as the reference condition with uncorrelated masker bands. In the other three conditions, the maskers consisted of a comodulated masker preceded by three different maskers: uncorrelated masker (RC), comodulated masker (CC), and the masker with comodulated flanking-bands (FC). The thick red line represents a tone that was presented with an IPD of 0 or π.

Our study consisted of three experiments: (i) psychoacoustical threshold measurements to quantify masking release induced by the combination of comodulation, IPD, and temporal context; (ii) intensity JND measurements to estimate the “perceived SNR” as a continuous measure; (iii) EEG experiments for measuring LAEPs as a neural correlate of the internal representation of the target signal in noise. For all three experiments, we used the same eight conditions. The conditions combined comodulation (uncorrelated/comodulated), interaural phase difference (0/π), and three different preceding maskers (random, comodulated, comodulated flanking bands) to induce masking release with comodulation, IPD, and temporal context (Figure 1). For the EEG experiment we used five, individually adjusted, levels of the target signal above masked threshold.

The stimulus consisted of five noise bands as a masker and a pure tone as a target signal (Figure 1A). One noise band was centered at the frequency of the target tone (center band, CB). The other bands were equally spaced with a distance of 120 Hz above and below the CB (flanking bands, FBs). Each masker band had a bandwidth of 20 Hz and a level of 60 dB SPL. The target tone was centered at 700 Hz. We chose this frequency setting to maximize the effect of the preceding stream formation on masking release based on previous work by Grose et al. (2009). Each interval consisted of a preceding masker with a duration of 500 ms (“preceding masker”) and a masked target tone with a duration of 200 ms (“masked tone interval”) (Figure 1A). We used four masker conditions. In the reference condition, the maskers had random intensity fluctuations across frequency for both the “preceding masker” and the “masked tone interval” (RR). In the three other conditions, the target tone was embedded in the following masker types: a masker with random intensity fluctuations across frequency (RC), a comodulated masker (CC), and a masker where only the FBs were comodulated (FC). For the RC condition, the preceding masker bands were random noise and would likely not be grouped into a stream. For the CC condition, the preceding masker bands might be grouped together to form one stream, facilitating the following target separation from the masker. For the FC condition, the preceding FBs might be grouped together and can be separated from the CB, impeding the following target separation from the masker. All maskers were presented diotically. The target tone was presented with an IPD of 0 (no BMLD)π (BMLD) in combination with the same four masker types, leading to a total of eight stimulus conditions.

All maskers had 20 ms raised-cosine on- and offset ramps. For the RC and FC conditions, the same on- and offset ramps were added in the transition between the preceding masker and the masked tone interval with a 50% overlap. The noise bands were generated in the frequency domain and transformed into the time domain. The noise bands were assigned numbers from a uniformly distributed random process to the real and imaginary parts of the respective frequency components. For the R masker, different numbers were assigned for each noise band. For the C masker, the same numbers were used for all five noise bands. The stimuli were generated with newly drawn numbers for each interval and each trial.

### 2.2. Apparatus

During all three experiments, the listeners were seated in a double-walled, soundproof booth. All stimuli were generated in MATLAB 2018b (TheMathworks, Natick, MA) with a sampling rate of 44,100 Hz and a 16-bit resolution, converted from digital to analog (RME Frieface UCX), amplified (Phonitor mini, SPL electronics), and played back through headphones (ER-2, Etymotic Research). The headphones were calibrated at the signal frequency of the tone. For the recording of AEPs, we used a g.Tec HIamp system with a sampling rate of 1,024 Hz. The 64 channels of active electrodes were set up with highly conductive electrode gel to reduce the impedance between the scalp and electrodes. The reference electrodes were placed close to the mastoid of both ears and the other electrodes were placed based on the g.GAMMAcap 64 channel setup from g.Tec.

### 2.3. Listeners

We recruited 15 normal-hearing listeners. None of them reported any history of hearing impairment. All but one listener had pure-tone hearing thresholds within 15 dB HL for the standard audiometric frequencies from 125 to 4,000 Hz. One listener had a threshold of 20 dB at 125 Hz. All participants provided written informed consent, and all experiments were approved by the Science-Ethics Committee for the Capital Region of Denmark (reference H-16036391). All of them participated in the first experiment, eleven of them participated in the second experiment, and ten of them participated in the third experiment.

### 2.4. Procedure

In the first experiment, we measured masked thresholds individually for the eight stimulus conditions presented in random order. We used an adaptive, three-interval, three-alternative forced-choice procedure (3-AFC) with a one-up, two-down rule to estimate the 70.7% of the psychometric function (Levitt, 1971; Ewert, 2013). Two intervals contained the masking noise only. The remaining interval contained the target tone in addition to the masker. The three intervals were presented with a temporal gap of 500 ms in between. The listeners' task was to select the interval with the target tone by pressing the corresponding number key (1, 2, 3) on the keyboard. Visual feedback was provided, indicating whether the answer was “WRONG” or “CORRECT”. The initial level of the target tone was set to 75 dB SPL and was adjusted with an initial step size of 8 dB. The step size was halved after each lower reversal until it reached the minimum step size of 1 dB. The signal level at a minimum step size of 1 dB was measured six times, and the mean of the last six reversals was used as the estimated threshold. Each listener performed three threshold measurements for all conditions. The average of three measurements was used as individual masked thresholds for the next two experiments. Additional measurements were performed if the thresholds from the last three measurements had a standard deviation larger than 3 dB.

In the second experiment, we measured intensity JNDs individually at six supra-threshold levels for all conditions. The intensity of the tone was individually adjusted for each listener to match levels of +0 dB (threshold), +5, +10, +15, +20, and +25 dB relative to the threshold. The individual mean of three threshold measurements from the first experiment was used to set the reference of +0 dB. We used the same setup and 3-AFC method as for the first experiment. Two intervals contained the masked target tone with a fixed level at one of the supra-threshold levels (“reference interval”), and the remaining interval contained the masked target tone with a higher level than the others (“target interval”). The intervals were presented with a temporal gap of 500 ms in between. Listeners were asked to select the interval with the tone of highest intensity by pressing the corresponding number key (1, 2, 3) on the keyboard. Visual feedback was provided, indicating whether the answer was “WRONG” or “CORRECT.” The order of conditions and supra-threshold levels were randomized. The initial level of the tone in the target interval was set to 75 dB SPL. The level of the target tone was adjusted with the initial step size of 8 dB. The step size was halved after each lower reversal until it reached the minimum step size of 1 dB. The signal level at a minimum step size of 1 dB was measured six times, and the mean of the last six reversals was used as the JND. Listeners were familiarized with the task by a test run. Each listener performed three trials for all conditions. If the supra-threshold level exceeded 80 dB, the intensity JND measure was skipped. We calculated the intensity JND by subtracting (in dB) the level of “reference intervals” from the minimum level of discriminable tone in “the target interval”.

In the third experiment, we measured late auditory evoked potentials (LAEPs) at three supra-threshold levels for all conditions. The intensity of the tone was individually adjusted for each listener to match levels of +15, +20, and +25 dB above the threshold. The individual mean of three threshold measurements from the first experiment was used to set supra-threshold levels. The stimuli for each condition and supra-threshold level were presented 400 times in random order. In addition, noise-only stimuli were presented 40 times for each condition. The presentations were separated by a random inter-stimulus interval of 500 ms with jitter. During the experiment, a silent movie with subtitles was presented on a low-radiation screen. The listeners were asked to sit comfortably and avoid movement as much as possible. The experiment was divided into six blocks of approximately 38 min each. These were divided into two sessions on different days.

### 2.5. Data analysis

#### 2.5.1. The threshold measurements

We calculated CMR and BMLD to quantify the amount of masking release in eight conditions. We used several acronyms for masking release measures for each condition as follows. For comodulation masking release (CMR),


(1) $$\begin{array}{l} \text{CM}{\text{R}}_{\text{m}/\text{ipd}}=\text{threshold}\left[\text{R}{\text{R}}_{\text{ipd}}\right]-\text{threshold}\left[{\text{m}}_{\text{ipd}}\right], \end{array}$$

Here, *m* stands for one of three masker types (RC, CC, FC) and *ipd* stands for the IPD of the tone between two ears (0 or π). As an example, *CMR*_*C*_*C*__π__ is the amount of a decrease in threshold in *CC*_π_ condition compared to *RR*_π_ condition. A positive value indicates a decreased detection threshold, and a negative value indicates an increased detection threshold. For binaural masking level difference (BMLD),


(2) $$\begin{array}{l} \text{BML}{\text{D}}_{\text{m}}=\text{threshold}\left[{\text{m}}_{0}\right]-\text{threshold}\left[{\text{m}}_{\text{π}}\right], \end{array}$$

As an example, *BMLD*_*CC*_ is the amount of a decrease in threshold in *CC* condition with IPD of π compared to *CC* condition without IPD (these two conditions would correspond to *N*_0_*S*_0_ and *N*_0_*S*_π_ conditions in a classical BMLD experiment). For statistical analysis, the Lilliefors test was used for a normality test. To compare CMR and BMLD across four masker types, one-way ANOVA followed by Tukey's multiple comparison tests were used. In the case where the data did not follow a normal distribution, the Kruskal–Wallis test was used, followed by Dunn's multiple comparison test. To compare CMR between two conditions with the same masker type but with different IPD, a Wilcoxon signed-rank test was used.

#### 2.5.2. The intensity JNDs and the perceived SNR

Given the complexity of quantifying salience, the present study used intensity JND at fixed intensities as a measure to derive “perceived SNR” (pSNR) as a continuous variable. As the primary parameter changed in the experiments was the intensity of the target tone, we used a measure related to intensity. We calculated the intensity JND of the target tone at a reference level (denoted Δ*L* in dB) by subtracting the level of the reference intervals (*L*) from the minimum intensity level of the discriminable tone in the target interval (*L* + Δ*L* in dB). The intensity JNDs were measured at discrete supra-threshold levels (e.g., at the threshold, +0, +5, +10, +15, +20, and +25 dB) for each listener. We then pooled all individual data for each condition, and fitted power functions.

Based on this fitted function, we could estimate Δ*L* at any level for each condition. This continuous fit was used to derive the pSNR(*L*) for each condition from 1 to 10 (arbitrary scale) as follows: The pSNR was defined to be 1 at the average threshold *L*_th_ across listeners. Then, the intensity JND at *L*_th_ (denoted Δ*L*_th_ in dB) was read out with help of the fitted function for the given condition. The intensity JND was added to the reference level (*L*_th_ in this case). This level was then assigned pSNR(*L*_ref_2__) of 2 and served as the reference for the next iteration. Hence, the algorithm was:


(3) $$\begin{array}{lll} \text{pSNR}\left(L_{{\text{ref}}_{n}}\right) & =n & \text{with }n=1,\ldots ,10 \end{array}$$

(4) $$\begin{array}{lll} L_{{\text{ref}}_{n}} & =L_{{\text{ref}}_{n-1}}+\Delta L_{{\text{ref}}_{n-1}} & \text{with }L_{{\text{ref}}_{1}}:=L_{\text{th}} \end{array}$$

We repeated this algorithm until the pSNR reached 10. In addition, we estimated the Weber fraction by dividing the intensity difference Δ*I* by the intensity of the target tone (*I*) for better comparison with data from the literature. We reported the Weber fraction on the logarithmic scale as 10*log*(Δ*I*/*I*).

#### 2.5.3. Late auditory evoked potentials (LAEPs)

Collected data were analyzed using FieldTrip (Oostenveld et al., 2011). In short, the EEG data were partitioned into epochs from −300 to 850 ms relative to the onset of the preceding masker. The region of interest was the central position (Cz), and the reference signals were the average of two electrodes near the mastoids. Each epoch was low-pass (Butterworth IIR filter, 6th order, zero-phase) filtered with a cut-off frequency of 20 Hz. Detrending, baseline correction, and weighted averaging were applied to increase the signal-to-noise ratio (Riedel et al., 2001). Trials containing signals exceeding 100 μV in any channel were rejected as artifacts. For auditory evoked potentials (AEPs), we extracted the signals from 100 ms before the onset of the target tone and 100 ms after the offset of the target tone from the averaged epochs. Baseline correction was applied considering a 100 ms pre-stimulus period. The grand mean of AEPs was computed with arithmetic mean over all individual AEPs. We selected the first negative component (N1) and the second positive component (P2) as a peak measure individually. We defined the peak of the first negative deflection in the time window between 100 and 200 ms (with respect to the target onset) as N1 and the peak of the second positive deflection in the time window between 200 and 300 ms as P2. This was estimated for each individual AEPs to eliminate individual differences in latency. Peak amplitudes were extracted by the MATLAB function *findpeaks* by locating minima and maxima within the time frame defined for N1 and P2, respectively. Extracted LAEPs were visually verified. In the case where multiple components were found, the one with the largest amplitude was selected. When there was no component found, this condition was excluded from the analysis.

## 3. Results

### 3.1. Experiment 1. Masked thresholds

**Figure 2:**
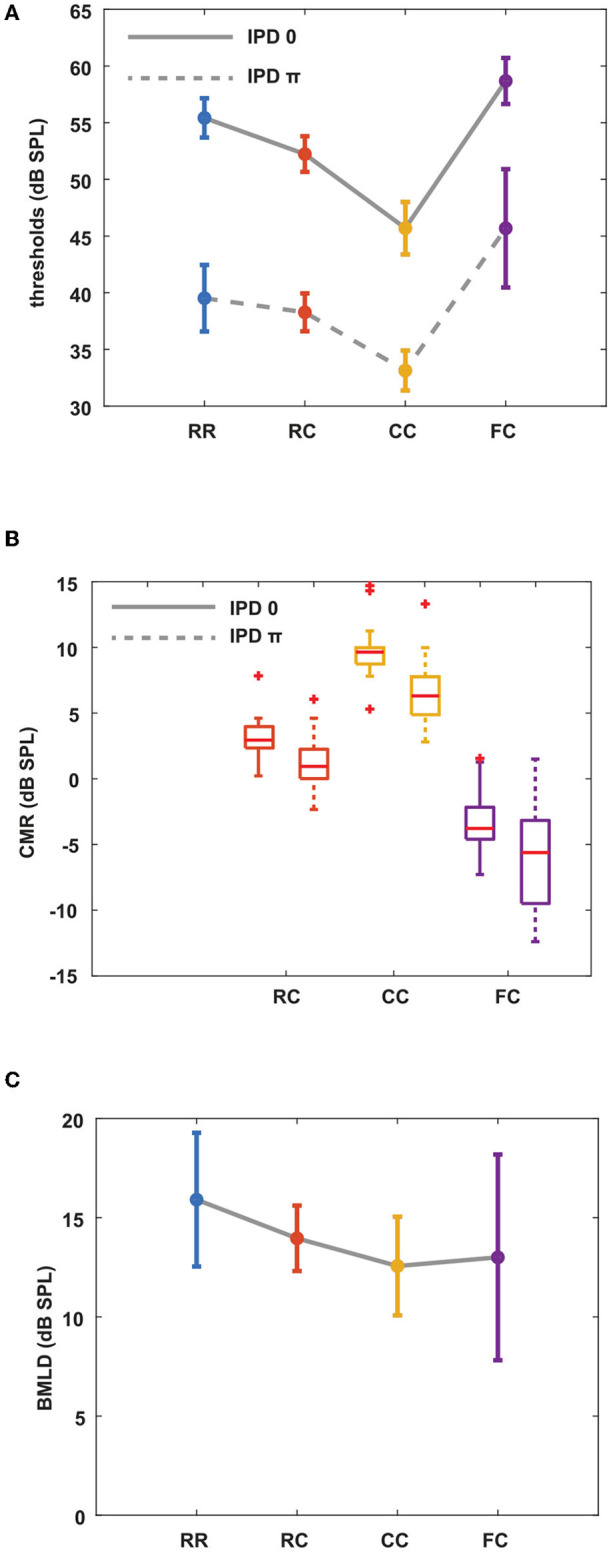
Mean masked thresholds for all conditions and masking releases. CMR and BMLD are calculated based on measured thresholds. Each color represents four masker types. Blue indicates RR condition, orange indicates RC condition, yellow indicates CC condition, and purple indicates FC condition. Solid lines represent diotic conditions (IPD of 0) and dotted lines represent dichotic conditions (IPD of π). Error bars indicate ± one standard deviation. **(A)** Masked thresholds from eight masking release conditions averaged over all listeners. **(B)** CMR with the RR masker as a reference. **(C)** BMLD for all masker types.

Our first question was how three auditory cues, comodulation, IPD, and temporal contexts will induce masking release. We designed four different masker types to investigate how preceding stream formation would affect following target detection in noise, together with comodulation and IPD cues. We measured masked thresholds for eight masking release conditions. Figure 2A shows the mean masked thresholds for the eight stimulus conditions. In diotic conditions, thresholds were highest for the FC condition and lowest for the CC condition. The observed mean threshold across all the participants for the *RR*_0_ condition was 55.4 dB. The *RC*_0_ condition had a mean threshold of 52.2 dB. In the *CC*_0_ condition, the threshold was 45.7 dB. In the *FC*_0_ condition, the mean threshold was 58.7 dB. Based on these threshold measures, we calculated CMR values for each condition by using the RR condition as reference [eq. (1)]. As shown in Figure 2B, these results show that the preceding stream formation can affect the following target detection, or CMR. *CMR*_*RC*0_ was 3.2 dB, *CMR*_*CC*0_ was 9.7 dB and *CMR*_*FC*0_ was -3.3 dB, which are in line with previous studies by Dau et al. (2005, 2009); Grose et al. (2009). As the CMR data did not follow a normal distribution (Lilliefors test), we used a Kruskal–Wallis test for statistical analysis. As the BMLD data followed a normal distribution, we applied the ANOVA test for statistical analysis. For *post-hoc* tests, we used Tukey's multiple comparisons for normal distributed data and Dunn's multiple comparisons for non-normal distributed data. Statistical analysis showed that CMR measures were different between different masker types. In diotic conditions, there was a significant difference in CMR between masker types (Kruskal–Wallis, *p* < 0.05). This is in line with the study by Grose et al. (2009), showing that the CMR was highest in CC condition and lowest in FC condition. This shows that preceding masker type can group masker bands into one stream (CC), facilitating the following target detection (large CMR), or separate masker bands into different stream (RC, FC), impacting the following target detection negatively (small or negative CMR).

The same overall pattern of the thresholds was found in the dichotic conditions. The *RR*_π_ had a mean threshold of 39.5 dB, and the *RC*_π_ condition had a mean threshold of 38.3 dB. In the *CC*_π_, the mean threshold was 33.1 dB, and that of the *FC*_π_ condition was 45.7 dB. We calculated CMR in the same manner as in diotic conditions. *CMR*_*RCπ*_ was 1.2 dB, *CMR*_*CCπ*_ was 6.4 dB and *CMR*_*FCπ*_ was −6.2 dB. Similar to diotic conditions, CMR was significantly different between masker types in dichotic conditions (Kruskal–Wallis, *p* < 0.05). Between diotic and dichotic conditions with the same masker type, all masker types showed a significant difference (Wilcoxon signed-rank test, *p* < 0.05). We further investigated whether the stream formation affected BMLD. Our hypothesis was that if the stream formation occurs at the level of A1, which in turn provides a feedback input to sub-cortical region, BMLD would be affected with preceding maskers as well. Figure 2C shows the BMLD calculated for each condition by using the threshold in the corresponding diotic condition as reference (Equation 2). *BMLD*_*RR*_ was 15.9 dB, *BMLD*_*RC*_ was 13.9 dB, *BMLD*_*CC*_ was 12.6 dB, and *BMLD*_*FC*_ was 13 dB. Multiple comparison tests showed that only the *BMLD*_*RR*_ and *BMLD*_*CC*_ differed significantly (one-way ANOVA, *p* < 0.05).

### 3.2. Experiment 2. Intensity JNDs

Our next goal was to estimate the pSNR with the intensity JND measures. Previous studies by Epp et al. (2013), Egger et al. (2019), suggest that the salience of the tone improves proportional to sensation level. In contrast to their data, our results show that the intensity-JND, and therefore pSNR, are more closely related to the physical target tone level than the sensation level.

**Figure 3:**
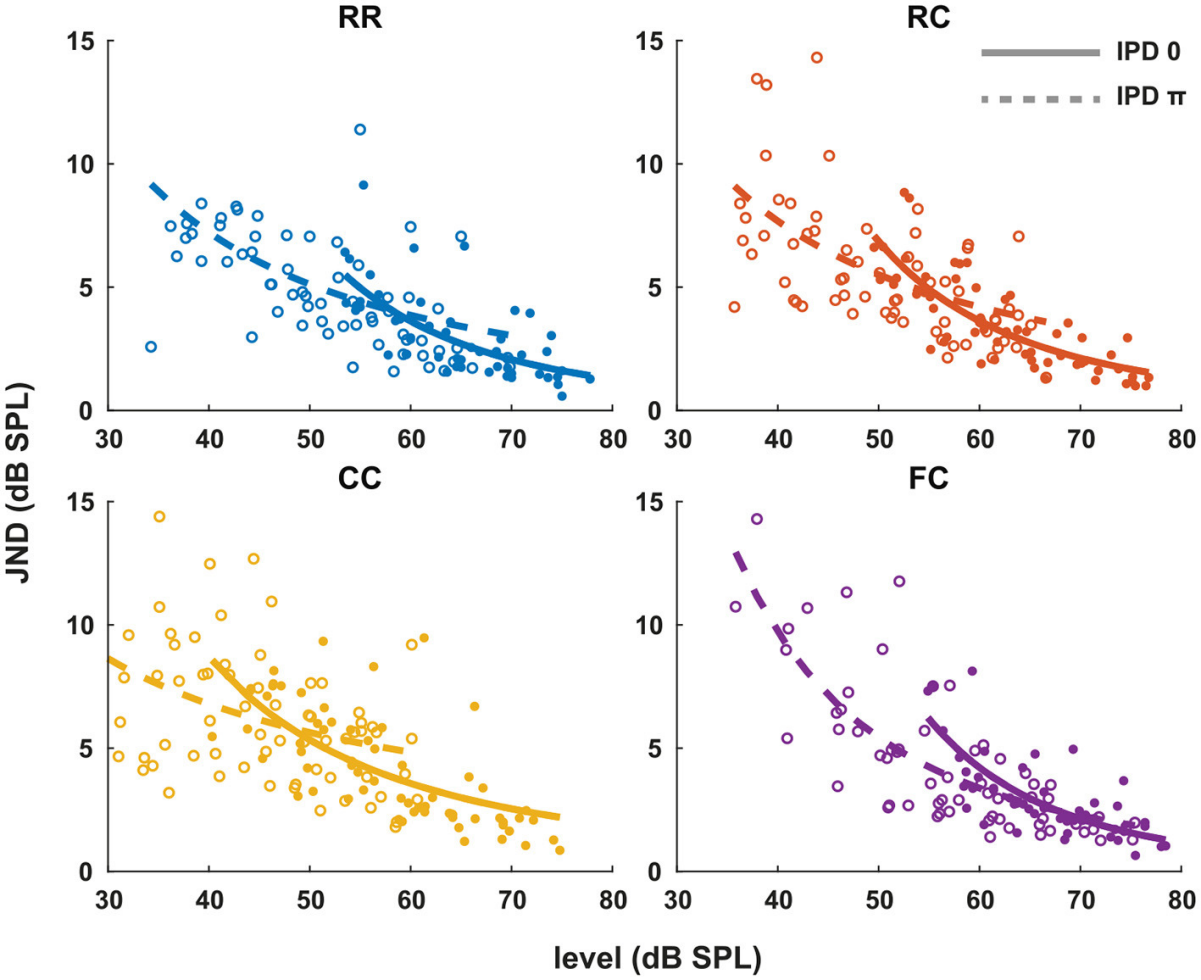
Intensity JNDs for all stimulus conditions and power law fit for diotic (solid line) and dichotic target signal (dashed line). Individual data are plotted as single points. The data for each condition are fitted with a power function. Each color represents four masker types. Blue indicates RR condition, orange indicates RC condition, yellow indicates CC condition, and purple indicates FC condition. Solid lines and filled circles represent diotic conditions (IPD of 0) and dotted lines and empty circles represent dichotic conditions (IPD of π). The goodness of fit for each condition with IPD of 0 is: RR (*R*^2^ = 0.4748), RC (*R*^2^ = 0.6614), CC (*R*^2^ = 0.4732), FC (*R*^2^ = 0.5604). The goodness of fit for each condition with IPD of π is: RR (*R*^2^ = 0.2743), RC (*R*^2^ = 0.1613), CC (*R*^2^ = 0.2946), FC (*R*^2^ = 0.6170).

**Figure 4:**
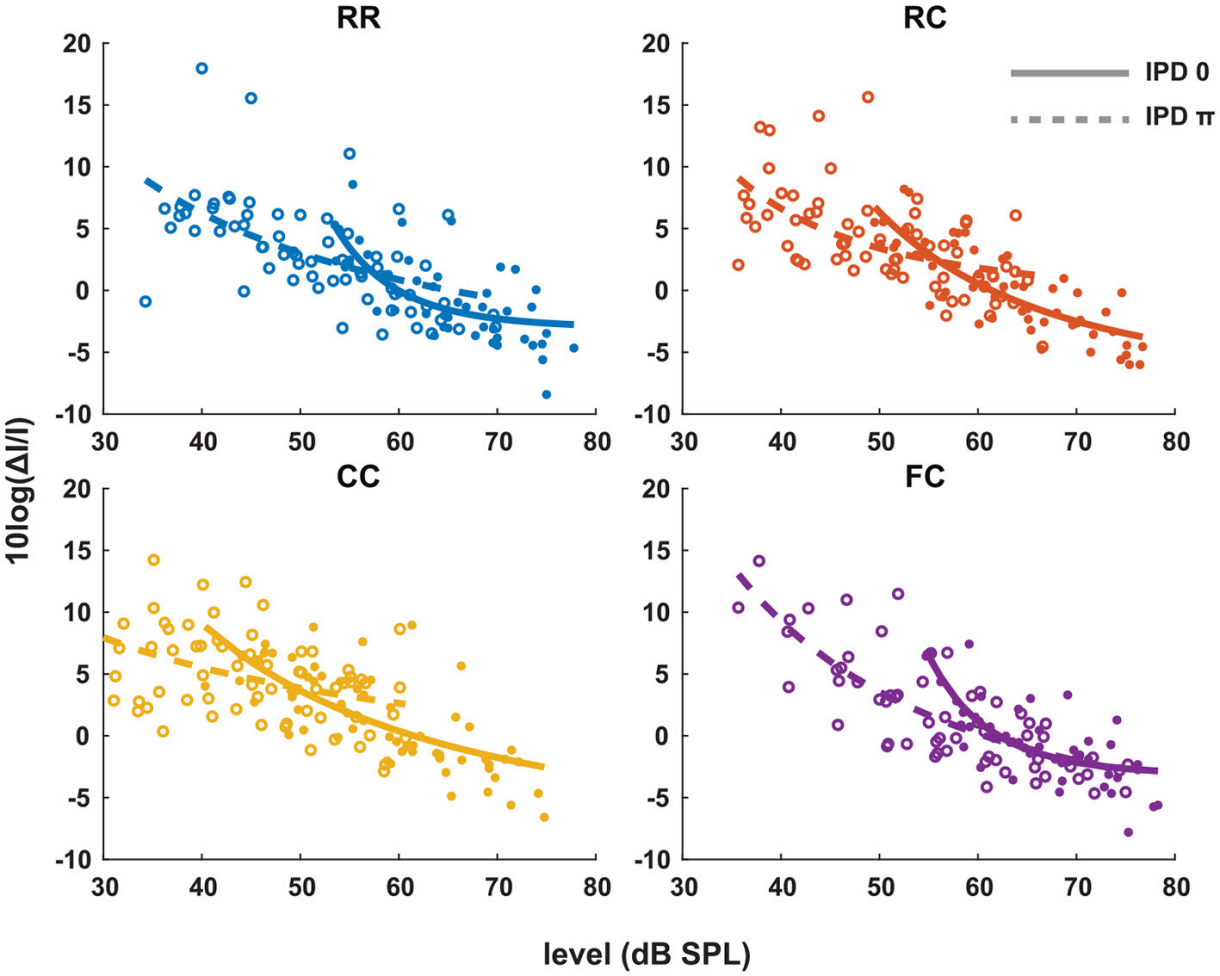
The Weber fraction of the intensity JND on a logarithmic scale. Individual data are plotted as single points. The data for each condition are fitted with a power function in the same manner as the Figure 3. Each color represents four masker types. Blue indicates RR condition, orange indicates RC condition, yellow indicates CC condition, and purple indicates FC condition. Solid lines and filled circles represent diotic conditions (IPD of 0) and dotted lines and empty circles represent dichotic conditions (IPD of π). The goodness of fit for each condition with IPD of 0 is: RR (*R*^2^ = 0.0.4819), RC (*R*^2^ = 0.6793), CC (*R*^2^ = 0.5377), FC (*R*^2^ = 0.0.5492). The goodness of fit for each condition with IPD of π is: RR (*R*^2^ = 0.3394), RC (*R*^2^ = 0.3155), CC (*R*^2^ = 0.0.1762), FC (*R*^2^ = 0.6354).

Each panel in Figure 3 shows the individual intensity JND measures at sensation levels ranging from +0 dB (re individual threshold) to +25 dB in four masker types with both IPD of 0 (solid line) and IPD of π (dashed line). Individual intensity JND measures followed the physical target tone level. A previous study showed that the intensity JND follows a power law (Ozimek and Zwislocki, 1996). To test if this relationship holds in masking release conditions, we fitted the pooled intensity JND measures with a power function. Additionally, we calculated the Weber fraction of the intensity JND measures on a logarithmic scale based on stimulus intensity as 10*log*(Δ*I*/*I*), and fitted with a power function (Figure 4).

**Figure 5:**
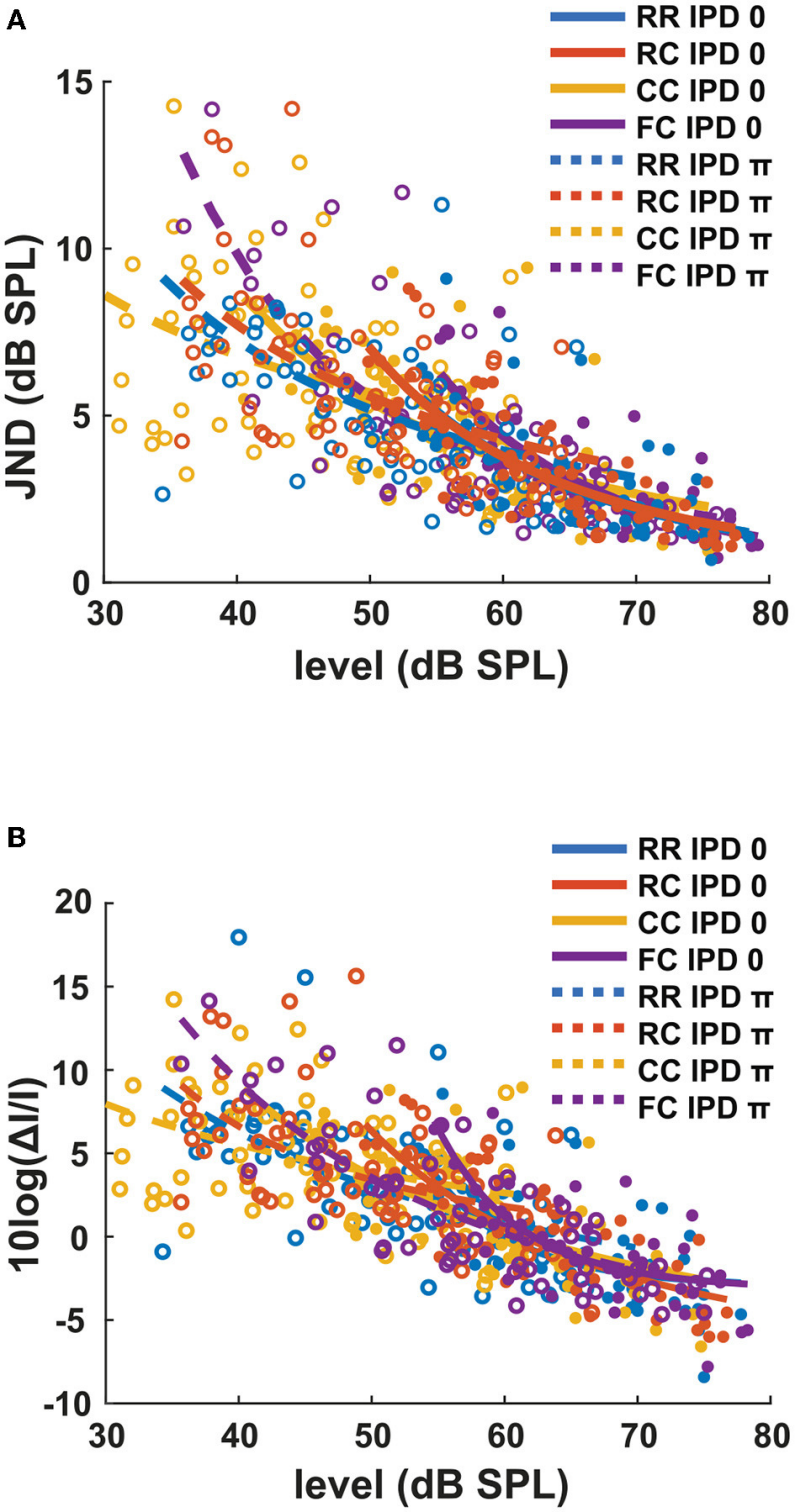
The intensity JND measures and the Weber fraction of the intensity JND in the logarithmic scale in all conditions. **(A)** The intensity JND measures. **(B)** The Weber fraction of the intensity JND in the logarithmic scale across all conditions. Each color represents four masker types. Blue indicates RR condition, orange indicates RC condition, yellow indicates CC condition, and purple indicates FC condition. Solid lines and filled circles represent diotic conditions (IPD of 0) and dotted lines and empty circles represent dichotic conditions (IPD of π).

In general, conditions with lower detection thresholds (e.g., *CC*_π_) showed larger JNDs compared to those with higher detection thresholds (e.g., *RR*_0_). To compare intensity JND measures between conditions, the intensity JND measures across all conditions were pooled together. Figure 5A shows the averaged intensity JND and Figure 5B shows the Weber fraction of the intensity JND on a logarithmic scale as a function of the physical target tone level in the reference signal. The intensity JND measures of all conditions and listeners are shown with scatter plots and fitted with a power function. The intensity JNDs decrease with increasing level of the target tone in all masking release conditions. This suggests that the pSNR depends on the target tone level rather than the supra-threshold level (see 4.3 for discussion). We estimated the goodness of fit for each condition and reported in the legend of each figure (Figures 3, 4). The Weber fraction of intensity JND in the logarithmic scale showed a comparable goodness of fit with the power function than the JND expressed as Δ*I*/*I*.

**Figure 6:**
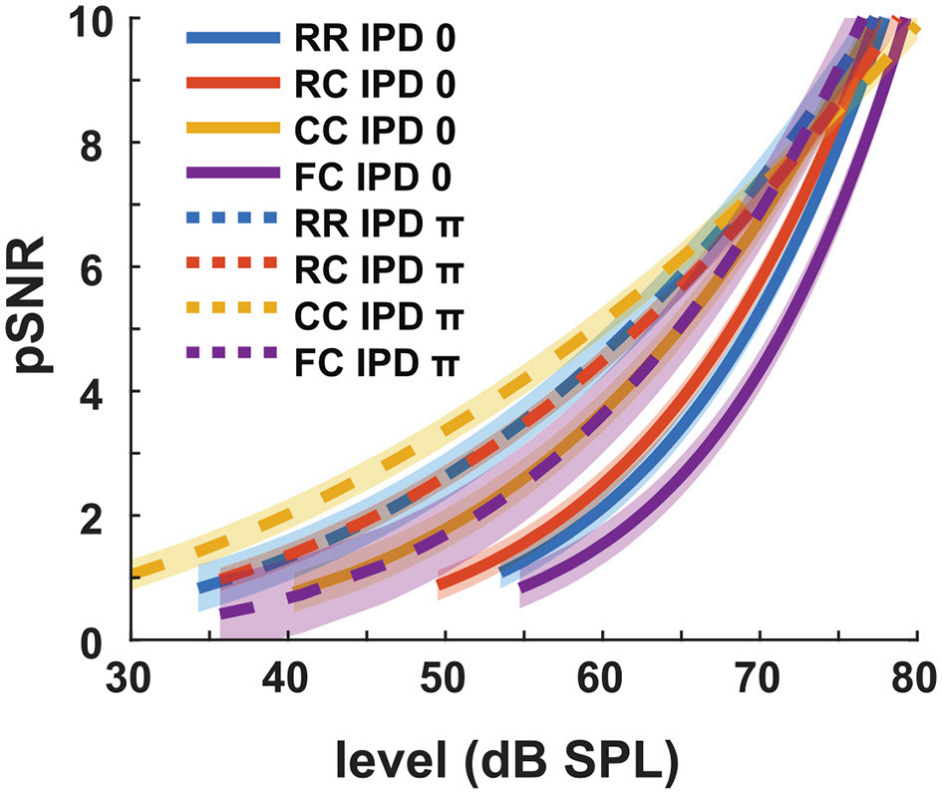
Estimated pSNR at individual intensity levels. Each color represents four masker types. Blue indicates RR condition, orange indicates RC condition, yellow indicates CC condition, and purple indicates FC condition. Solid lines represent diotic conditions (IPD of 0) and dotted lines represent dichotic conditions (IPD of π). The shaded areas indicate ± one root mean square error.

We define pSNR in the context of this study as the perceptual quantity that describes how clearly the tone is perceived in noise. We arbitrarily defined that the pSNR increases by one when the target tone level is increased by the intensity JND. The estimated pSNR is shown in Figure 6. At the same physical target tone level, the pSNR was higher for conditions with lower detection thresholds. For instance, the pSNR was higher in dichotic conditions compared to diotic conditions with the same masker type. It converged for the different conditions when the target tone level was above 70 dB SPL.

### 3.3. Experiment 3. Late auditory evoked potentials

**Figure 7:**
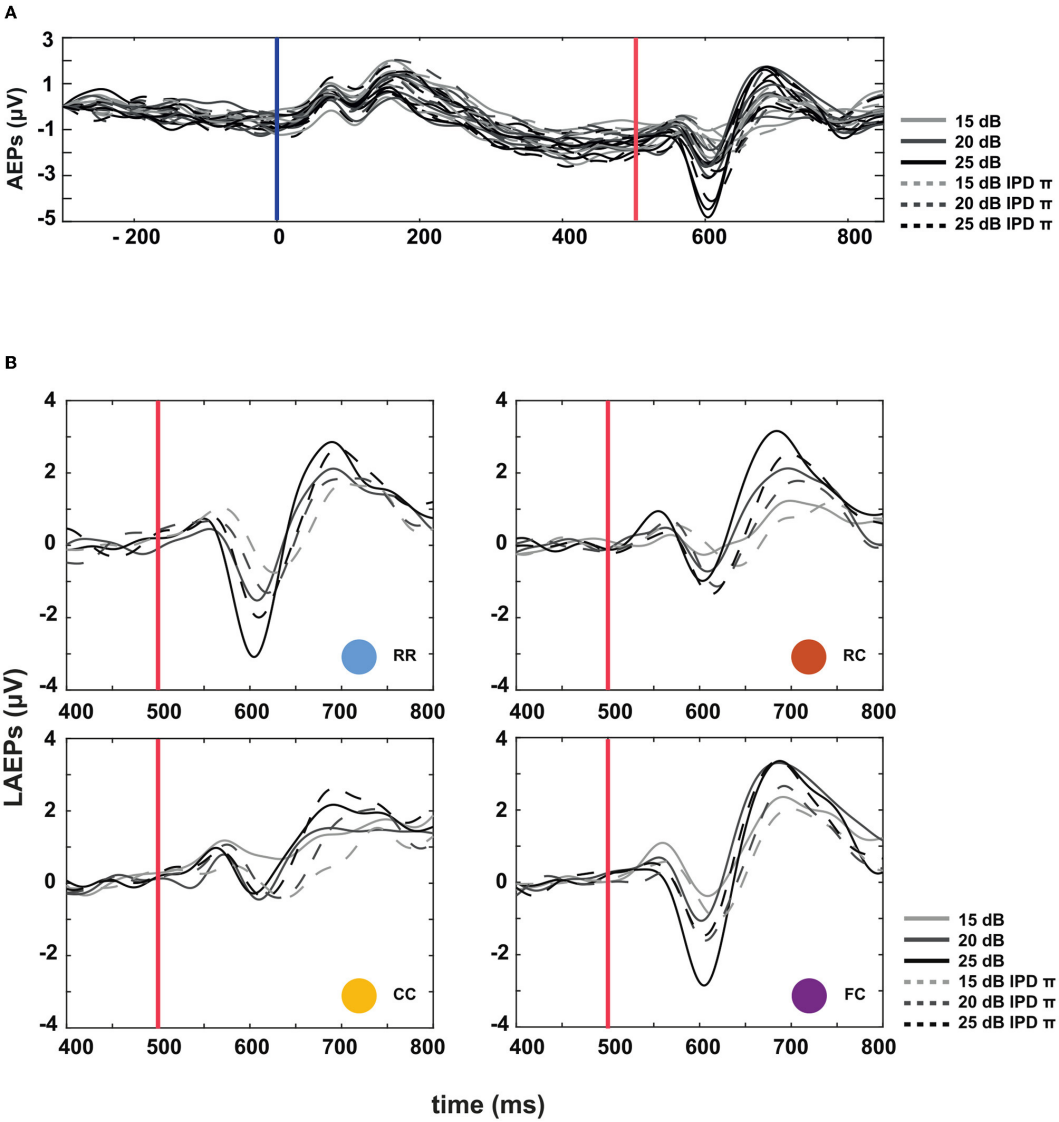
Auditory evoked potentials (AEPs) at three supra-threshold levels: +15, +20, and +25 dB. **(A)** AEPs averaged over all listeners. Masker onset is at *t* = 0, target tone onset at *t* = 500 ms. Solid lines represent the four masker types with diotic target signals, and the dotted lines represent the four masker types with dichotic target signals. **(B)** Late auditory evoked potentials (LAEPs) to the target tone in the time interval ranging from 400 to 800 ms post masker onset.

**Figure 8:**
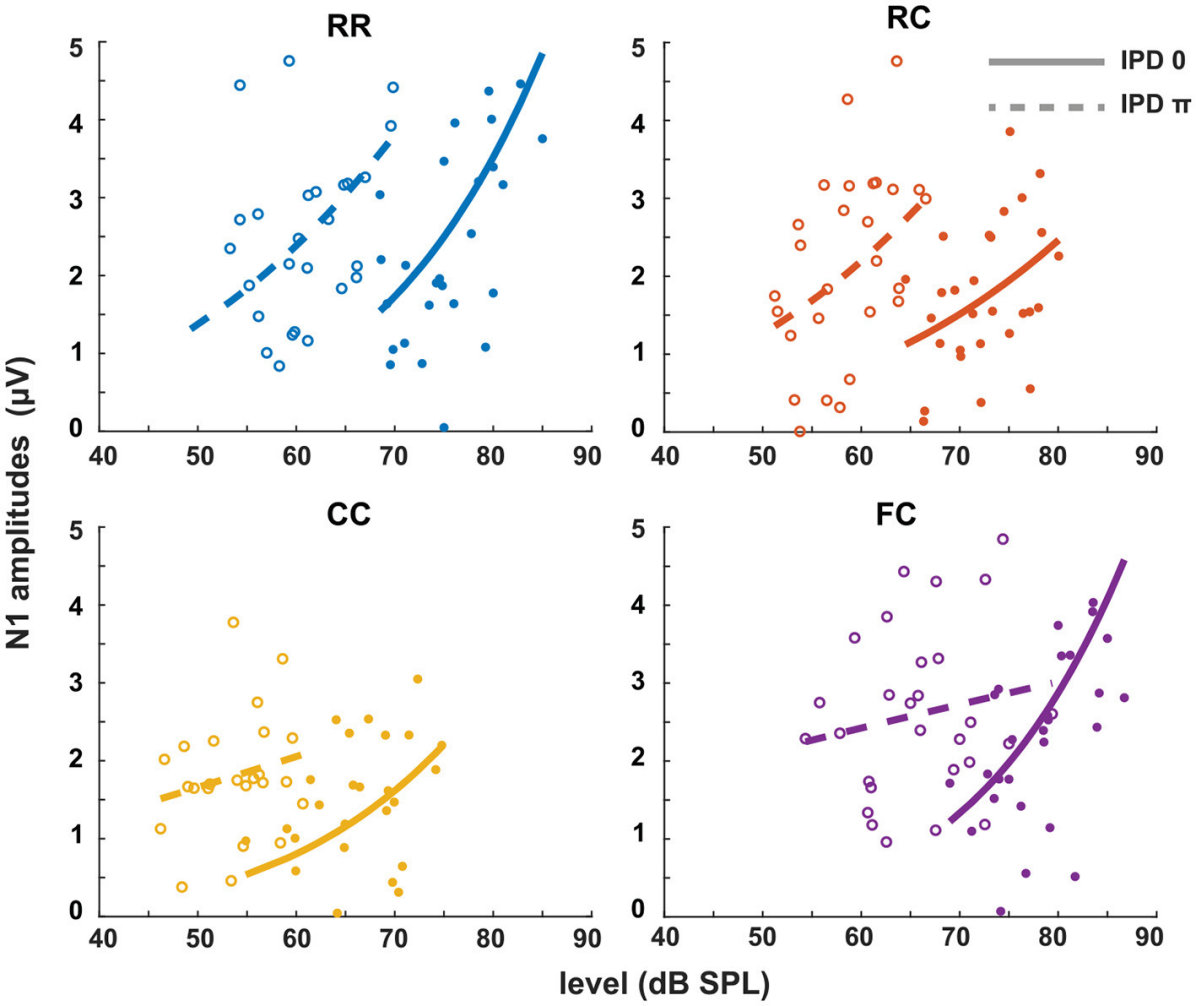
N1 amplitudes as a function of target tone level at supra-threshold levels: +15, +20, and +25 dB. Individual data are plotted as single points. The data for each condition are fitted with a power function (line). Blue represents the RR condition, orange the RC condition, yellow the CC condition, and purple the FC condition. The solid lines represent the data of IPD 0 and the dotted line the data of IPD π. For each condition, the goodness of fit (*R*^2^) with IPD of 0 is: RR (*R*^2^ = 0.2776), RC (*R*^2^ = 0.1657), CC (*R*^2^ = 0.2179), FC (*R*^2^ = 0.2893). The goodness of fit with IPD of π is: RR (*R*^2^ = 0.2075), RC (*R*^2^ = 0.1390), CC (*R*^2^ = 0.0469), FC (*R*^2^ = 0.0288).

**Figure 9:**
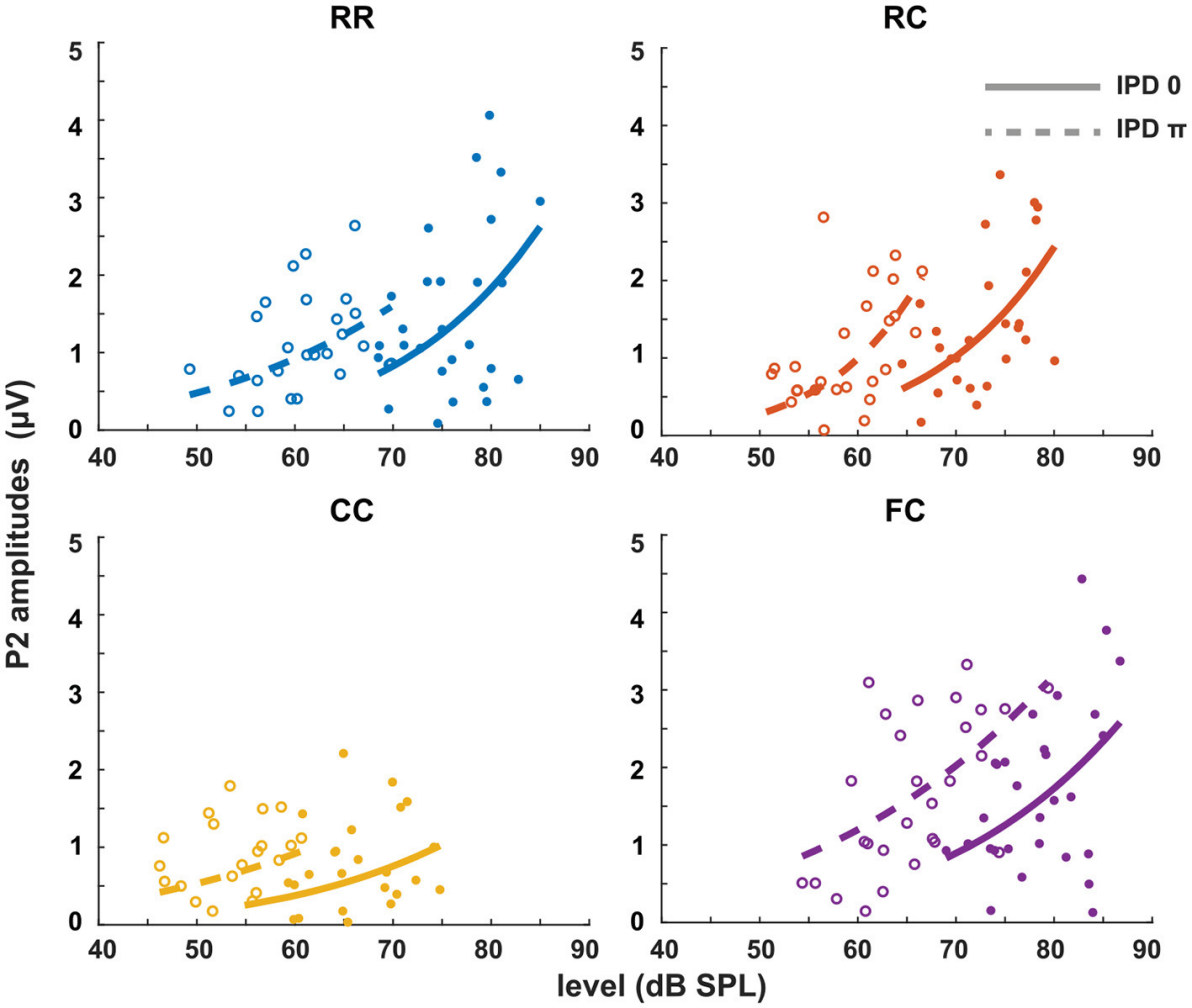
P2 amplitudes as a function of target tone level at supra-threshold levels: +15, +20, and +25 dB. Individual data are plotted as single points. The data for each condition are fitted with a power function (line). Blue represents the RR condition, orange the RC condition, yellow the CC condition, and purple the FC condition. The solid lines represent the data of IPD 0 and the dotted line the data of IPD π. For each condition, the goodness of fit with IPD of 0 is: RR (*R*^2^ = 0.1820), RC (*R*^2^ = 0.3253), CC (*R*^2^ = 0.0970), FC (*R*^2^ = 0.1646). The goodness of fit with IPD of π is: RR (*R*^2^ = 0.1847), RC (*R*^2^ = 0.3224), CC (*R*^2^ = 0.0601), FC (*R*^2^ = 0.3161).

**Figure 10:**
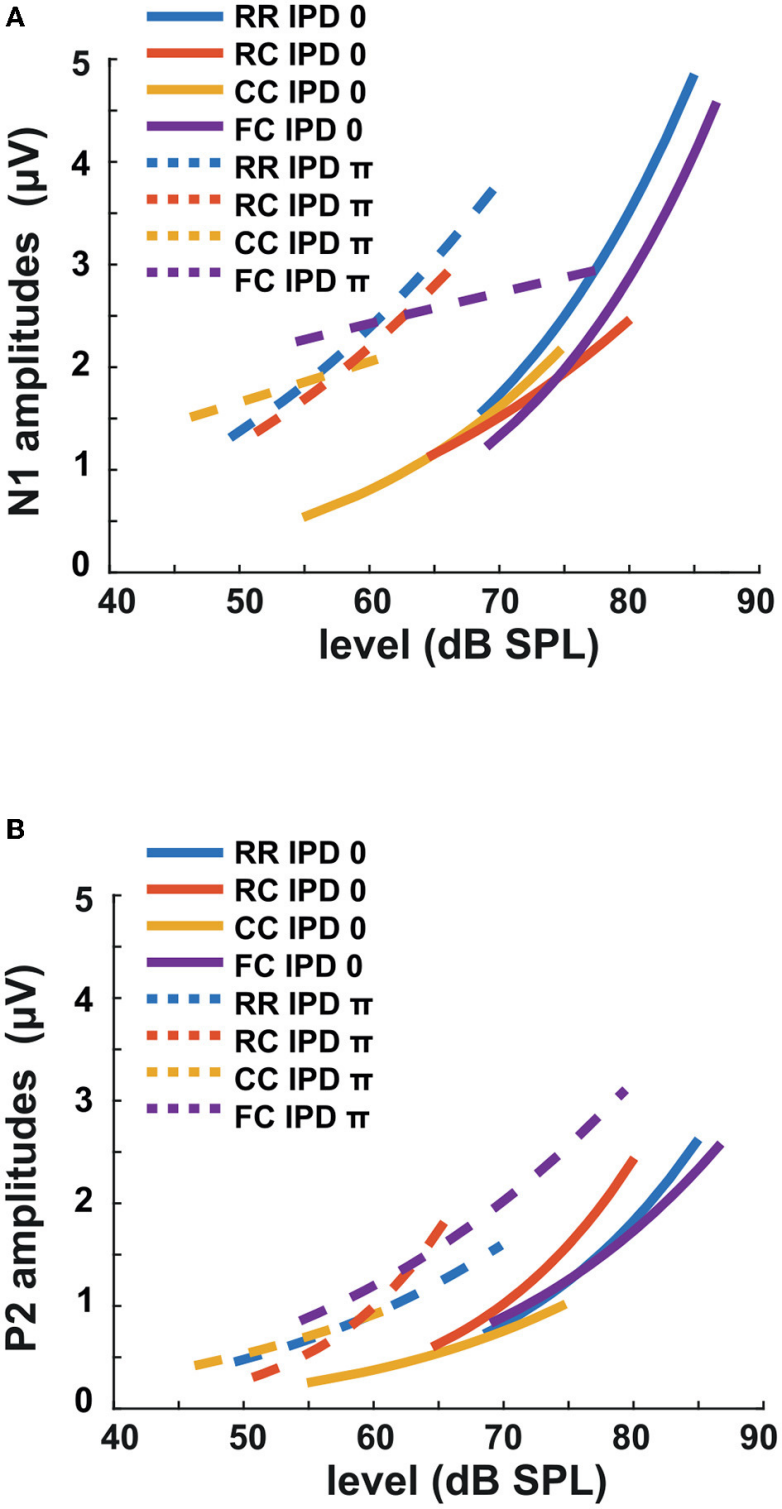
Amplitudes of LAEPs with a function of target tone level. Blue corresponds to RR condition, orange to RC, yellow to CC, and purple to FC condition. Solid lines represent the data of IPD 0, and dotted lines represent the data of IPD π. **(A)** N1 amplitudes with a function of target tone level. **(B)** P2 amplitudes with a function of target tone level.

**Figure 11:**
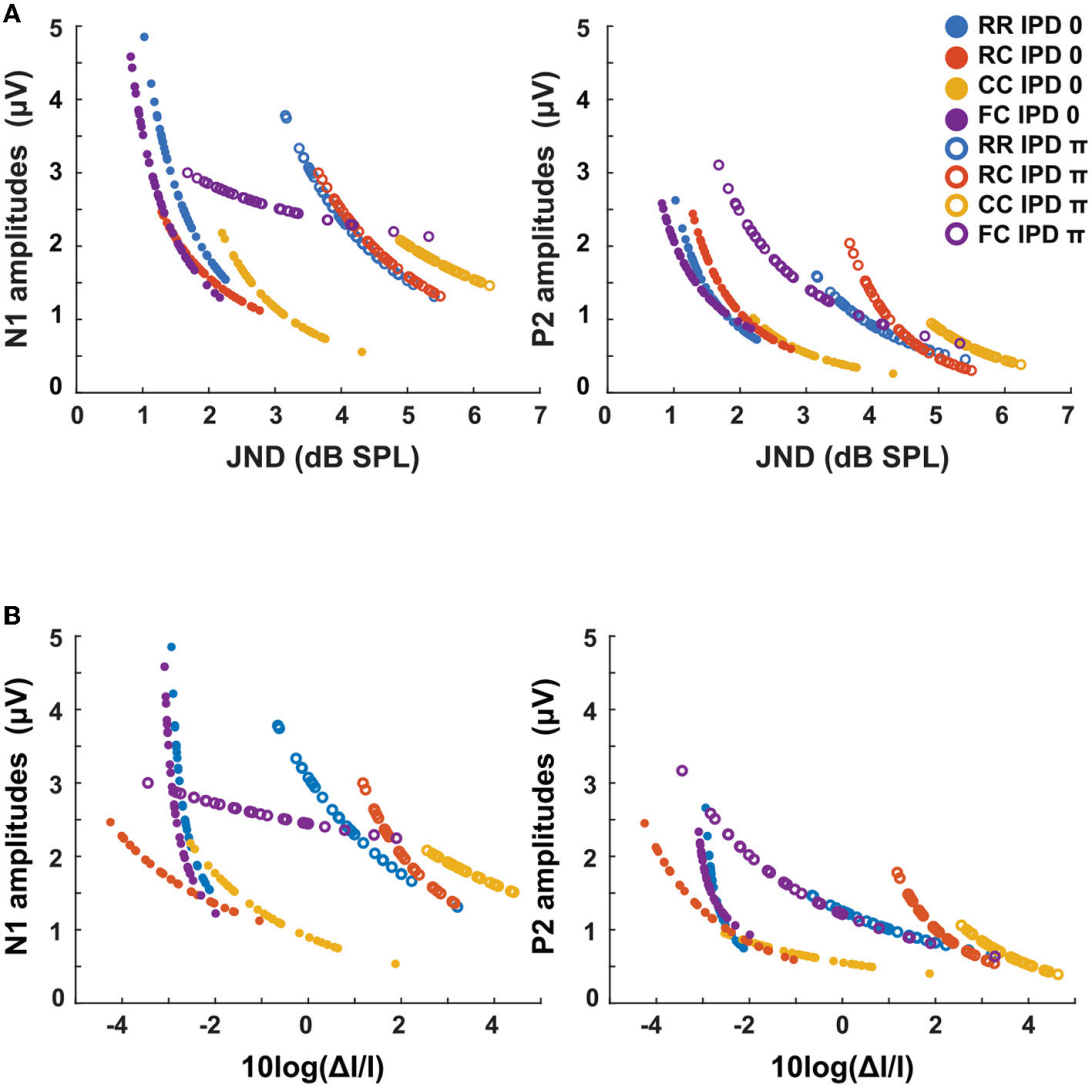
LAEPs as a function of intensity JND measures. The blue line represents the *RR* condition, the orange line the *RC* condition, the yellow line the *CC* condition, and the purple line the *FC* condition. The solid line represents the data for IPD of 0 and dotted lines the data for IPD of π. **(A)** LAEPs as a function of intensity JNDs in dB SPL. The data of N1 (left) and P2 (right) are fitted with a power function. **(B)** LAEPs as a function of the Weber fraction (10logΔ*I*/*I*). The data of N1 (left) and P2 (right) are fitted with a power function.

**Figure 12:**
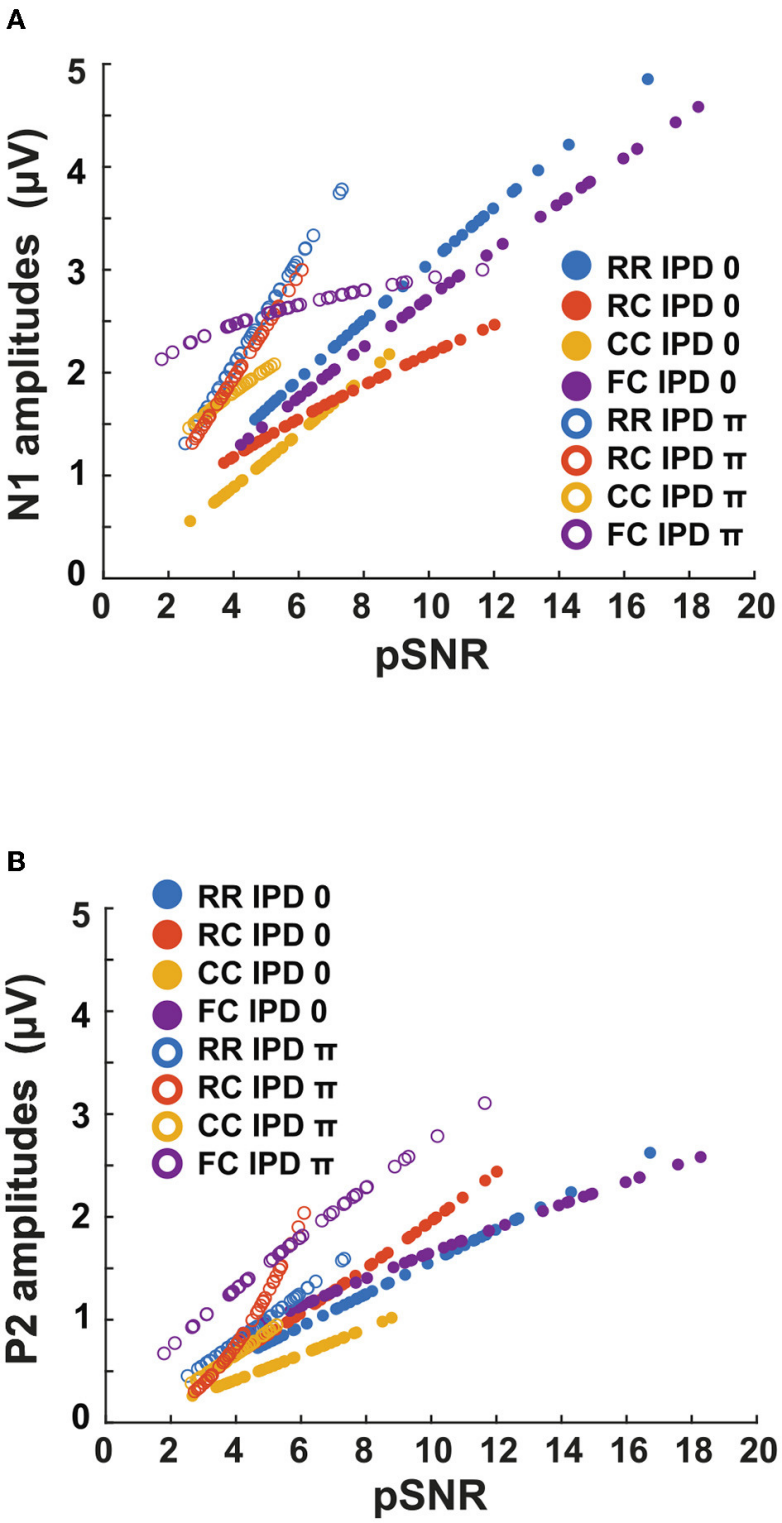
Estimated pSNR correlated with LAEP amplitudes. The blue line represents the *RR* condition, the orange line the *RC* condition, the yellow line the *CC* condition, and the purple line the *FC* condition. The filled circles represent the data for IPD of 0 and empty circles represent the data for IPD of π. **(A)** The N1 amplitudes with a function of estimated salience. **(B)** The P2 amplitudes with a function of estimated pSNR.

The third experiment aimed to investigate whether LAEPs can be used as a neural measure of the pSNR. Figure 7A shows the grand mean AEPs across all listeners for each condition. The plot shows the AEPs to diotic signals (solid lines) and dichotic signals (dashed lines) in the four masker types *RR*, *RC*, *CC*, and *FC*, respectively. Following the presentation of the stimuli (Figure 7A, blue line), an onset response was elicited, which went back to a constant value after around 300 ms post-onset. The response to the target signal was found from around 550 ms. Figure 7B shows the mean of LAEPs across all listeners for each condition. A characteristic LAEP wave morphology was found for all masker types with a small positive deflection (P1), followed by a large negative deflection (N1) and a large positive deflection (P2). We extracted the amplitudes of N1 and P2 individually. Then, we fitted N1 and P2 amplitudes as a function of the target tone level as shown in Figures 8, 9, respectively. The goodness of fitness is reported in each figure legend. Each panel shows the LAEPs of each masker type with both diotic (solid lines) and dichotic (dashed lines) target tones. All data were pooled in Figure 10 for better comparison. Here, both components showed an increase in amplitudes with increasing levels. The amplitudes of N1 showed more separation between diotic and dichotic conditions compared to the amplitudes of P2. However, the *FC*_π_ condition showed a diverting pattern. This may be related to the high variance in detection thresholds as observed in the first experiment. If the pSNR is stimulus-driven, or the result of bottom-up processing, only the quality of physical properties of the stimulus determines the behavioral outcome (Huang and Elhilali, 2017). If the detection performance was affected by other perceptual modalities (e.g., top-down attention), the N1 amplitudes may not be able to capture the enhanced pSNR by the IPD cue. Compared to the amplitudes of N1, the amplitudes of P2 showed better goodness of fit. With the addition of the IPD cue of π in the tone, the amplitude of P2 was also enhanced, indicating that the neural representation of the target tone was enhanced by the IPD cue. As shown in Figures 11A, B, the LAEPs as a function of the intensity JND show that the increase rate of P2 amplitudes is inversely correlated to the magnitude of the intensity JND. To directly link the pSNR and the LAEPs, we pooled the pSNR and amplitudes of N1 and P2 (Figure 12). P2 amplitudes have a more coherent relationship with pSNR than N1 amplitudes.

## 4. Discussion

### 4.1. Effect of preceding maskers on CMR and BMLD

The results of the first experiment (Figure 2A) showed that the effect of preceding stream formation on CMR is large compared to the one on BMLD. In both diotic and dichotic conditions, the effect of preceding maskers on CMR was similar, as shown in Figure 2B. The amount of CMR (Figure 2B) was highest for the condition where the masker was comodulated for the whole duration of the interval (*CC*). CMR was reduced if the preceding masker had uncorrelated intensity fluctuations across frequency (*RC*). CMR was negative in the condition where the comodulation of the preceding masker only spanned the FBs (*FC*). In a previous study by Grose et al. (2009), when the target tone was preceded and followed by maskers, similar results were found. This is also consistent with studies where the reduction of CMR by preceding or following stream formation was suggested as high-level auditory processing (Dau et al., 2005, 2009). They interpreted the results as the temporal effect of preceding and following stream formation. Even though the stimuli in the present study had no following masker after the offset of the target tone, the thresholds were in line with the results in Grose et al. (2009) with both preceding and following maskers in a CMR paradigm. This suggests that the preceding masker plays a strong role in inducing auditory streams, which may impede the following stream formation by comodulation. This also can be strongly supported by physiological study by Sollini and Chadderton (2016) and in a psychoacoustical study by Sollini et al. (2022). With similar conditions, they also showed a comparable effect size of the preceding masker on following target detection and a temporal built-up during object-formation. In addition, CMR was significantly reduced in dichotic conditions (e.g., *CC*_0_ vs. *CC*_π_). This is also in line with previous studies by Schooneveldt and Moore (1989), Cohen and Schubert (1991), Ernst and Verhey (2006), Epp and Verhey (2009).

While the effect of preceding masker on CMR was strong, its effect on BMLD was less pronounced. The amount of BMLD (Figure 2C) was similar across conditions and only showed a significant difference between the RR and the CC condition. The BMLD in the *CC* condition was lower by 3.3 dB compared to the *RR* condition. A potential reason for reduced BMLD could be that the overall improvement of the target signal by comodulation and IPD reached a maximum. A similar phenomenon was observed in Epp and Verhey (2009) where listeners with a high BMLD showed slightly reduced CMR. Interestingly, the *FC* condition showed high individual variability in detection thresholds when the tone was presented with an IPD of π. In dichotic conditions, some listeners reported that they could hear the target tone when only the noise was presented. This may indicate that the FBs and the CB might have been separated into different objects by comodulated FBs in the preceding masker. This may induce a tone-like perception of the CB as the noise bandwidth was as narrow as 20 Hz. This may have contributed to low CMR and BMLD. For listeners with high CMR and BMLD, we speculated that actively focusing on the IPD cue facilitated target tone detection (top-down attention). However, this needs to be further investigated.

If preceding maskers can form an auditory object or stream, prior knowledge can affect following sound perception or masking release. Physiological evidence shows that neural correlates of comodulation processing can be found as early as the CN level (Pressnitzer et al., 2001; Neuert et al., 2004), while there is broad consensus that binaural information is processed at levels past the CN like in the medial- and lateral superior olive (MSO, LSO) and the IC (e.g., Shackleton et al., 2003, 2005; Tollin and Yin, 2005; Day and Semple, 2011; Zohar et al., 2011). Under the assumption of bottom-up processing of the masked signal along the auditory pathway, beneficial auditory cues enhance the internal representation of the target signal at the brainstem level (CN, MSO, LSO, IC), inducing masking release. Hence, we hypothesized that if the effect of the preceding maskers triggers additional high-level auditory processing, this will reduce the combined CMR and BMLD. In this study, the data suggest that BMLD is hardly affected by preceding maskers, while CMR varies strongly dependent on the type of preceding masker. This is not in agreement with the interpretation that the effect of preceding masker on masking release as the result of high-level auditory processing (e.g., temporal integration). A possible explanation is the influence of the cortical feedback where priming to the preceding noise at the level of A1 has an influence on the processing of sensory information at the brainstem (Asilador and Llano, 2021). In this scenario, the auditory system uses accumulated information of incoming sound, which can be understood as adaptation at a “system level”. This adaptation at the cortical level could affect auditory processing at the brainstem. Such an auditory efferent system from the auditory cortex to the CN could explain the effect of preceding maskers on CMR but not BMLD (Terreros and Delano, 2015). Another possibility could be that even though CMR and BMLD are measures of masking release, only the processing of comodulation, but not the processing of binaural cues, contribute to object- and stream formation.

Lastly, one might also speculate about the role of adaptation processes at the peripheral level in the effect of preceding maskers. Similar to the paradigm used in this study, various psychophysical and neural phenomena have shown the influence of preceding signals on the following target tone perception, termed as “auditory enhancement” (e.g., Nelson and Young, 2010; Kreft et al., 2018). In these studies, the preceding maskers were broadband noise with a spectral notch around the target signal. The presence of a spectral gap around the signal frequency in the preceding masker enhanced the target detection. The underlying mechanism of “auditory enhancement” has been attributed to the adaptation at both low- and high-level auditory processing. For supporting the adaptation at low-level auditory processing, Kreft et al. (2018) suggested that olivocochlear efferents may induce the adaptation effect in a longer time scale than the auditory nerve fibers (Guinan, 2006). If this is the case, how modulation patterns (e.g., *RR*, *RC*, *CC*, and *FC*) result in different degrees of CMR reduction is in question. Based on a physiological study where modulation pattern encoding was found at the CN level, the connectivity between the CN and the medial olivocochlear (MOC) efferents may play a role (Pressnitzer et al., 2001; Oertel et al., 2011). However, further psychoacoustic and physiological studies are needed to develop these ideas.

### 4.2. Neural intensity encoding at supra-threshold levels

The second experiment (Figure 3) showed that the intensity JND was inversely proportional to the physical sound level rather than the level above masked threshold. This is consistent with data from the literature for pure tones in quiet (e.g., Ozimek and Zwislocki, 1996) where the intensity JND decreased according to the power function of sensation level. As shown in Figure 4, expression of the JND on a relative scale to the reference intensity [10*log*(Δ*I*/*I*)] showed almost no dependence of the JND on the masker type (*RR*, *RC*, *CC*, *FC*) and the IPD (0, π). This means that, for a given target tone level, regardless of the difference in masked thresholds, the intensity JND on a relative scale was the same. Such level dependency of JND is interesting in terms of the supra-threshold level. Between two conditions, for instance, the supra-threshold levels can differ by up to 25 dB at a target tone level of 70 dB (*FC*_0_ vs. *CC*_π_, Figure 2A). Still, for both cases, the same relative amount of intensity increment was required for the discrimination (Figure 5).

This poses a question on the neural encoding of sound intensity. Sound intensity is often assumed to be encoded by spike rate (Cai et al., 2009; Micheyl et al., 2013b). However, auditory nerve fibers (ANFs) show rather a sigmoid function of spike rates respective to sound intensity (Bruce et al., 2018). Therefore, if the intensity JND measures are the result of rate-based encoding, an additional mechanism must exist to combine information across ANFs (Viemeister, 1988). Several studies have suggested that the auditory cortex plays such a role in intensity discrimination (Dykstra et al., 2012; Micheyl et al., 2013a). We propose that such a mechanism could also be located at the level of the CN. Physiological studies found neural correlates of CMR where neural activity was affected by comodulation (e.g., Nelken et al., 1999; Pressnitzer et al., 2001; Neuert et al., 2004). At a given stimulus level, the neural activity is higher in conditions with a masking release compared to a condition without a masking release. In this case, it seems plausible that the internal representation of the tone rather than the physical target tone level is relevant for sound perception. However, as the intensity JND is the same for the same target tone level regardless of the amount of masking release, our results indicate that the physical target tone level is encoded and preserved, in addition to the enhanced neural representation at thresholds as an internal signal-to-noise ratio (iSNR). For the intensity encoding at the level of CN, small cells showed preserved intensity encoding of the target tone in the presence of the noise (Hockley et al., 2022). These cells displayed a unique rate-level function where the spike rate increases without saturation with increasing levels up to 90 dB SPL (Hockley et al., 2022). This could be a possible mechanism of the intensity coding in masking release conditions.

### 4.3. Estimation of perceived SNR with the intensity JND

We estimated the pSNR based on intensity JND measurements. The pSNR at the threshold was arbitrarily set to one. This was based on the idea that the detection of a signal by the auditory system is possible once the internal representation of that signal exceeds a critical iSNR. In the previous studies, both salience and P2 amplitudes were analyzed as the function of supra-threshold levels rather than physical levels (e.g., Epp and Verhey, 2009; Egger et al., 2019). Their interpretation was based on a linear signal theory approach, stating that any addition of signal energy above the detection threshold should increase the iSNR proportionally with the increase of the signal intensity. That is, the salience rating would increase proportional to the supra-threshold levels, at least close to threshold. The data from the present study are not in line with these studies. As shown in Figure 6, the pSNR increases as a function of the target tone level, but each condition shows different slopes. This is contradictory to the linear signal theory approach to iSNR as our data show that the change in pSNR is dependent on the physical target tone level rather than on the sensation level. At higher intensities, the pSNR converge, indicating the vanishing effect of the beneficial cues leading to CMR and BMLD. This converging behavior might indicate that, at high signal-to-noise ratios, the perceptual quality of the tone is hardly affected by the presence of a low-intensity noise. This may further indicate that various auditory cues, such as comodulation, IPD, and temporal contexts, are effectively used when the physical SNR is not high enough to induce a high pSNR.

From a physiological point of view, this interpretation would imply that the physical target tone level needs to be encoded, along with and improvement of the neural representation of the signal through comodulation, IPD, and temporal contexts. This clearly outlines a shortcoming of the simplified model by Epp and Verhey (2009). In this simplified model, the output of a wideband inhibition stage for each ear served as the inputs of an interaural cancellation stage. In this model, superposition was interpreted as the summation of the effect of comodulation and IPD in dB. This model did not include any non-linearity that would explain the behavioral outcome of the current study. Thus, further studies are needed to enable us to extend this argument toward more complex signals like speech. Furthermore, it should be highlighted that the pSNR in the present study and the salience used in the study by Egger et al. (2019) likely reflect different aspects of perception. Egger et al. (2019) suggested that some listeners might have used a partial loudness cue to assess the salience of the presented target tone. This is consistent with the present study in terms of the dependence on the physical target tone level rather than the level above the masked threshold. However, with existing loudness models, the relation between pSNR (salience) and loudness growth as a function of the target tone level in masking release conditions is unclear.

### 4.4. LAEPs and intensity JNDs

In the present study, we estimated the increase of P2 amplitudes as a function of the target tone level. As shown in Figure 10A, N1 amplitudes showed a more prominent difference between diotic and dichotic conditions than P2 amplitudes. However, the *FC*_π_ showed a smaller correlation with target tone levels than other conditions. On the other hand, P2 amplitudes were proportional to the target tone level in all conditions and showed higher goodness of fit than N1 amplitudes (Figure 10B). If N1 amplitudes reflect BMLD processing at the IC level, the data on P2 amplitudes might suggest an additional higher-level BMLD processing. P2 amplitudes were larger in dichotic conditions than in diotic conditions, suggesting enhanced iSNR. Between conditions with the same IPD, the difference was marginal compared to the behaviorally estimated pSNR.

We hypothesized that LAEPs are linked to the internal neural representation of intensity JNDs (Figure 11A). We estimated both LAEPs and the intensity JND from the fitted function of LAEPs and intensity JNDs. We used individual supra-threshold levels of all conditions from fifteen listeners as input to the fitted functions. The amplitudes of LAEPs were inversely correlated with the intensity JND measures and the Weber fraction (Figure 11B). P2 amplitudes showed a steeper increase as the intensity JND decreases. With the Weber fraction of the intensity JND, which showed a better correlation with the target tone level, P2 amplitudes had a linear relationship to the Weber fraction of the intensity JND. P2 amplitudes across conditions with the same IPD were less diverted from each other compared to the intensity JND measures.

### 4.5. LAEPs and perceived SNR

To investigate if P2 amplitudes could be a neural measure for pSNR, we estimated pSNR at the supra-threshold levels individually (+15, +20, +25 dB) by using intensity JNDs. The use of intensity JNDs might be justified in this context as intensity contributes to salience (Kaya et al., 2020) and thereby to the overall perception of the sound, and was the parameter changed in the EEG experiment. Our initial hypothesis was that pSNR is linked to iSNR which can be reflected in the P2 component of the LAEP. This would result in the same P2 amplitude for the same pSNR (Figure 12B), but different curves for the P1 amplitudes.

Figure 12 shows the relation between the pSNR and the amplitudes of N1 and P2. Although P2 amplitudes were more correlated with pSNR than N1 amplitudes, the results showed deviating patterns in dichotic conditions (e.g., *RC*_π_, *FC*_π_). Hence, the initial hypothesis could not be supported. In the first experiment, CMR and BMLD showed a non-linear interaction, such as reduced CMR in dichotic conditions and high variability of CMR and BMLD in the FC dichotic conditions. In the second experiment, the intensity JND showed high variance for low target levels. As the pSNR was based on the intensity JND measures, this might have affected the accuracy of the pSNR. Therefore, pSNR estimation based on the Weber fraction of the intensity JND measures may produce a more robust prediction. However, the validity of translating the Weber fraction of the intensity JND to the pSNR needs to be further investigated.

In the third experiment, N1 amplitudes were not correlated with audibility, or BMLD processing, in the *FC*_π_ condition. This also suggests a possible higher-order auditory processing that may play a role in shaping neural responses. As the neural mechanisms underlying such non-linearity are unclear, further physiological evidence is needed to make a clear conclusion on to what extent P2 amplitudes can reflect the auditory processing stages and predict the pSNR or even salience. If additional high-level auditory processing is involved in combining CMR and BMLD with temporal integration, AEPs that elicited later than P2 (e.g., P300) might provide more insights on the feasibility of electrophysiological measures for the perception of a masked tone in noise.

The understanding of auditory salience is still challenging, and it remains to be shown if pSNR is related to salience. Various studies clearly showed the complexity behind a clear definition of the salience of sounds. For example, Soeta and Ariki (2020) investigated properties of bird sound that modulate their ability to be separated in an urban environment. They identified various features including measures of pitch strength, apparent source width, and amplitude fluctuations. Kaya et al. (2020) evaluated the salience of sounds using a combined listening and EEG paradigm. They found that various features including pitch, timbre, and intensity can modulate salience, and there is a non-linear interaction of these attributes that change neural power and phase in the EEG signal. While these studies focused largely on sounds that are clearly audible (i.e., well above the threshold), the present study focused on low intensities to investigate the relevance of masking release from the near threshold level to high above the threshold levels. The stimuli used in this study were simplistic compared to the sounds used in the previous studies (e.g., bird songs, speech, music). Hence, the use of the term “perceived SNR” in this study was to imply the notion that various sound attributes can contribute to the perception of a target in noise, and that the neural representation of the target in noise can be modulated by changes of these sound attributes (Kaya et al., 2020). In this regard, our study can be a stepping stone for investigating the perceptual attributes with both behavioral and electrophysiological measures, and future studies can go forward to include more complex stimuli to account for realistic auditory scene analysis such as speech in noise.

## 5. Conclusion

In this study, we investigated the detection and discrimination of masked tones in masking release conditions. Auditory cues such as comodulation, IPD, and temporal contexts, could enhance the detection performance when the physical SNR is low. Interestingly, at supra-threshold levels, the discrimination performance was highly dependent on the physical target tone level. Regardless of the masking release conditions, the intensity JND measures were similar at the same physical sound intensities. Furthermore, the estimated pSNR was higher with additional auditory cues, together with lower detection thresholds compared to the conditions with fewer auditory cues. When the physical SNR is high enough, however, pSNR seems to converge to the same value across conditions, indicating the saturation of the perceptual quality of the sound. Lastly, the P2 amplitudes were more correlated with the behavioral measure of pSNR than the N1 amplitudes.

## Data availability statement

The raw data supporting the conclusions of this article will be made available by the authors, without undue reservation.

## Ethics statement

The studies involving human participants were reviewed and approved by Science-Ethics Committee for the Capital Region of Denmark. The patients/participants provided their written informed consent to participate in this study.

## Author contributions

Both authors listed have made a substantial, direct, and intellectual contribution to the work and approved it for publication.
